# Cytological Features of Clear Cell Sarcoma in Exfoliative and Effusion Cytology Specimen—A Case Report and Literature Review

**DOI:** 10.1002/dc.70164

**Published:** 2026-06-20

**Authors:** William H. Wu, Joanna K. M. Ng, Joshua J. X. Li

**Affiliations:** ^1^ Department of Pathology School of Clinical Medicine, the University of Hong Kong Pokfulam Hong Kong

**Keywords:** clear cell sarcoma, cytology, effusion, exfoliation

## Abstract

Clear cell sarcoma of soft tissue (CCSST) with malignant effusion is a rare yet clinically aggressive appearance, which may pose a diagnostic pitfall with its overlapping cytomorphologic features with metastatic melanoma and carcinoma. In this article, we reported a case with metastatic clear cell sarcoma of soft tissue with positive cytological findings in the sputum, as well as the peritoneal fluid and pericardial fluid. The cytological findings were compared and contrasted with cases reported in the literature. Malignant effusion cytology, variable positivity of melanocytic immunocytochemistry markers (namely S100, SOX10, Melan‐A, HMB‐45) and confirmatory molecular pathology techniques (e.g., detection of underlying EWSR1 gene rearrangement by fluorescent in situ hybridization or by reverse transcription polymerase chain reaction for EWSR1‐ATF1 gene fusion) formed the key diagnostic approach of this entity.

## 1 Introduction

Clear cell sarcoma of soft tissue (CCSST) is a rare, malignant mesenchymal neoplasm of uncertain differentiation, first described by Enzinger in 1965 as “clear cell sarcoma of tendon and aponeuroses” [1]. It shows a predilection to deep soft tissue plane of distal extremities, with a peak incidence in the thirties and forties and featuring a slight female preponderance [2, 3]. Histologically it is characterized by nests or fascicles of plump spindle or ovoid cells possessing palely eosinophilic or clear cytoplasm and prominent nucleoli, at times accompanied by melanin pigment. By immunohistochemistry, the tumour cells may express different melanocytic markers, namely S‐100, SOX‐10, HMB‐45, and Melan‐A [4, 5]. Modern molecular pathology demonstrates the hallmark t(12;22) (q13;q12) reciprocal translocation in the majority of CCSST cases, resulting in the fusion of the *EWSR1* gene with the *ATF1* gene and formation of the pathogenic EWSR1‐ATF1 chimeric fusion protein [4]. Rare subsets of CCSST exhibit a variant t(2;22) (q34;q12) translocation, resulting in an EWSR1‐CREB1 fusion [6].

Due to the rarity of the entities, metastatic mesenchymal tumour involving effusion fluid, such as angiosarcoma [7] and malignant phyllodes tumour [8], poses significant diagnostic challenges in routine service. Metastatic CCSST in effusion fluid is no exception. We reported a case of metastatic clear cell sarcoma of soft tissue with positive cytological findings in the sputum, pericardial and peritoneal effusion. We also reviewed the literature and summarized the reported exfoliative and fine needle aspiration cytology, immunohistochemical and molecular pathology findings to formulate a cytological diagnostic approach of this rare yet aggressive neoplasm. To our best knowledge, it is the second post‐mortem CCSST case with positive effusion cytology finding reported in literature (Table 1).

**TABLE 1:** Summary of cases in the literature.

	Case	Age	Sex	Primary	Effusion cavity	Time to effusion	Treatment	Outcome (after effusion)
Current report	1	46	M	Right forearm	Peritoneal, pericardial, pleural, sputum	29 years after diagnosis	Excision and radiotherapy	DOD at 2 months
Parwani et al.	2	50	M	Left third toe/right mid‐thigh	Pericardial	3 years after diagnosis	Excision and chemotherapy	Unknown
Su et al.	3	Middle‐aged	F	Unknown	Pericardial and pleural	2 months after diagnosis	Unknown	Unknown
Creager et al.	4	53	F	Left knee	Pleural	Unknown	Unknown	Unknown
	5	15	F	Left thigh	Pleural	Unknown	Unknown	Unknown
	6	23	F	Left forearm	Pelvic	Unknown	Unknown	Unknown
	7	43	M	Lung	Pleural	Unknown	Unknown	Unknown
Caraway et al.	8	54	M	Right thumb	Pleural, bronchial, sputum	Unknown	Unknown	DOD at 53 months
Nguyen et al.	9	40	M	Right thigh	Pleural	3 year after diagnosis	Excision and chemotherapy	DOD at 38 months
	10	19	F	Left knee	Pleural	1 year after diagnosis	Excision and chemotherapy	DOD at 17 months
Keller et al.	11	Unknown	M	Penis	Pleural	Unknown	Unknown	Unknown

## 2 Case Presentation

**FIGURE 1:**
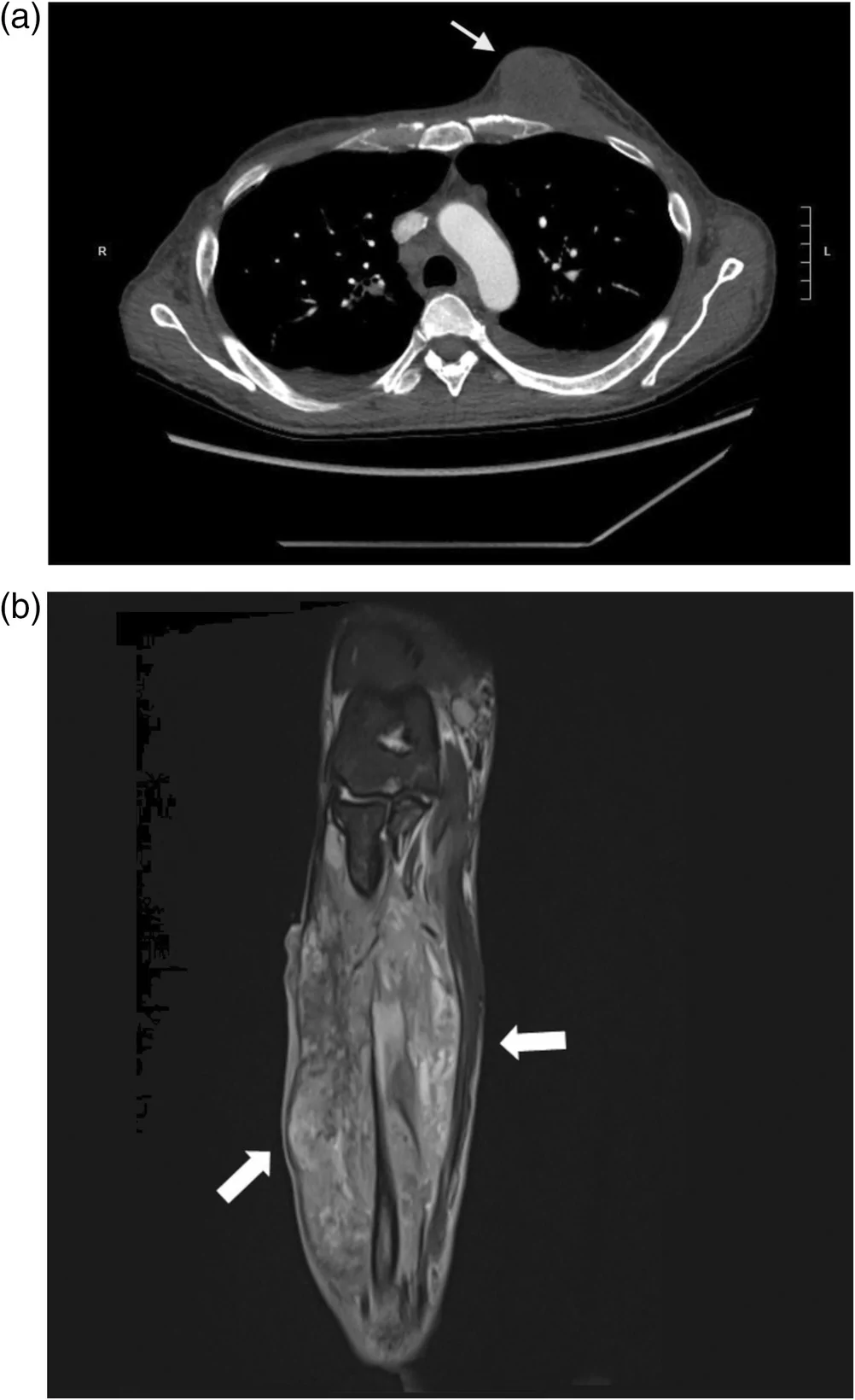
Radiological images of the right forearm and chest wall mass. (a) The CT showed a 2.8 cm heterogeneously enhancing soft tissue nodule (arrow) in the left medial upper chest wall, involving the subcutaneous layer and pectoralis muscle. (b) The MRI showed extensive heterogeneous T2 hyperintense enhancing infiltrative intramuscular mass involving both flexor and extensor compartments of the forearm (arrow).

The patient was a 46‐year‐old man with a history of right forearm clear cell sarcoma excised 30 years ago. He refused chemotherapy and received neoadjuvant radiotherapy. For the current episode he was admitted for suspected right forearm cellulitis with erythema, oedema and tenderness for 1 week, as well as left chest wall mass for 2 weeks. Ultrasound examination at admission showed extensive tenosynovitis‐like changes in the proximal right forearm, spanning 20 cm. It also showed a 3.3 cm irregular heterogeneous mass in the left anterior chest wall. The patient subsequently underwent contrast computed tomography (CT) of the thorax, abdomen and pelvis, and contrast magnetic resonance imaging (MRI) of the forearm and elbow. The CT showed a 2.8 cm heterogeneously enhancing soft tissue nodule in the left medial upper chest wall, involving the subcutaneous layer and pectoralis muscle (Figure 1a). The MRI showed extensive heterogeneous T2 hyperintense enhancing infiltrative intramuscular mass involving both flexor and extensor compartments of the forearm (Figure 1b).

**FIGURE 2:**
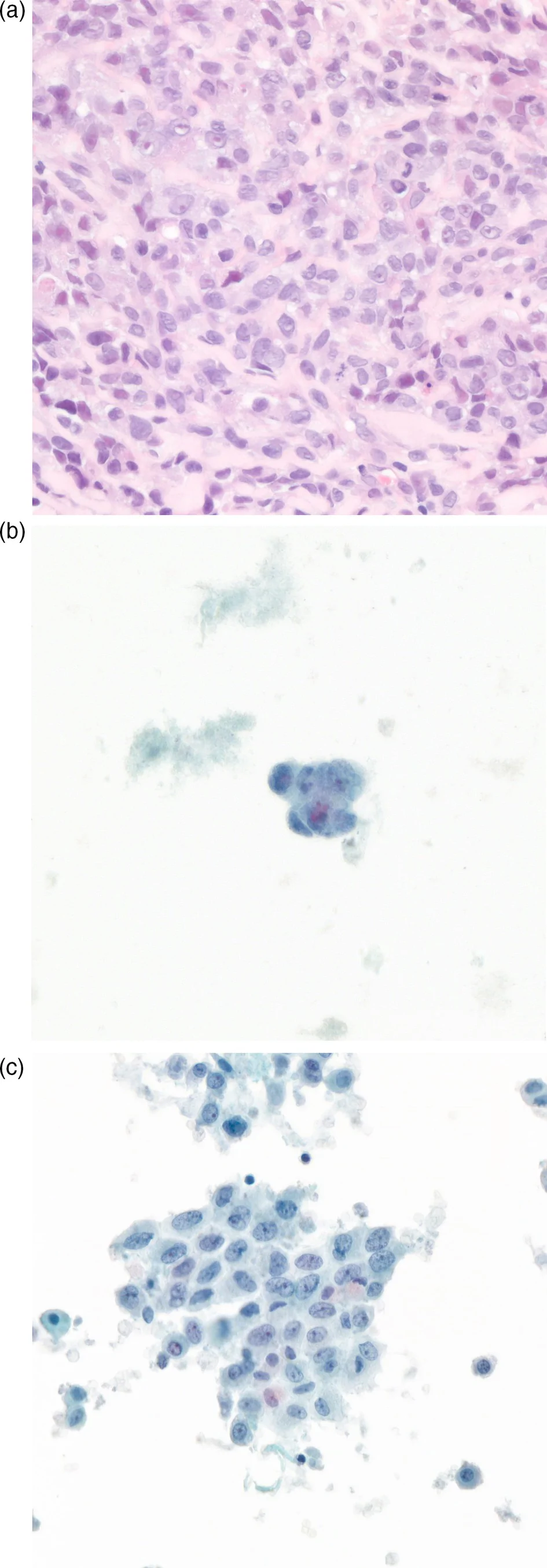
Biopsy and cytology specimens. (a) Core biopsy, 40×, H&E. (b) Sputum cytology, 40×, Pap stain. (c) Pericardial fluid cytology, 40×, Pap stain. [Color figure can be viewed at wileyonlinelibrary.com]

Ultrasound‐guided core biopsy of the chest wall lesion was performed. The biopsy showed nests, cords and isolated tumour cells possessing small amounts of eosinophilic cytoplasm, enlarged, irregular, moderately pleomorphic nuclei with prominent nucleoli. Melanin pigment was inconspicuous (Figure 2). By immunohistochemistry, the tumour cells were diffusely positive for S‐100 (Dako, Z0311) and SOX‐10 (Cell Marque, 383R‐16), and negative for HMB‐45 (Dako, M634). Reverse Transcription Polymerase Chain Reaction (RT‐PCR) on RNA extracted from the tumour‐rich area (80% tumour cellularity) of the section from the formalin‐fixed paraffin‐embedded block revealed the presence of EWSR1::ATF1 translocation products. DNA sequencing of the RT‐PCR products confirmed the presence of in‐frame translocations involving “exon 7 of the *EWSR1* gene and exon 4 of the *ATF1* gene” and “exon 8 of the *EWSR1* gene and exon 4 of the *ATF1* gene”. The diagnosis of involvement by clear cell sarcoma of soft tissue was made.

**FIGURE 3:**
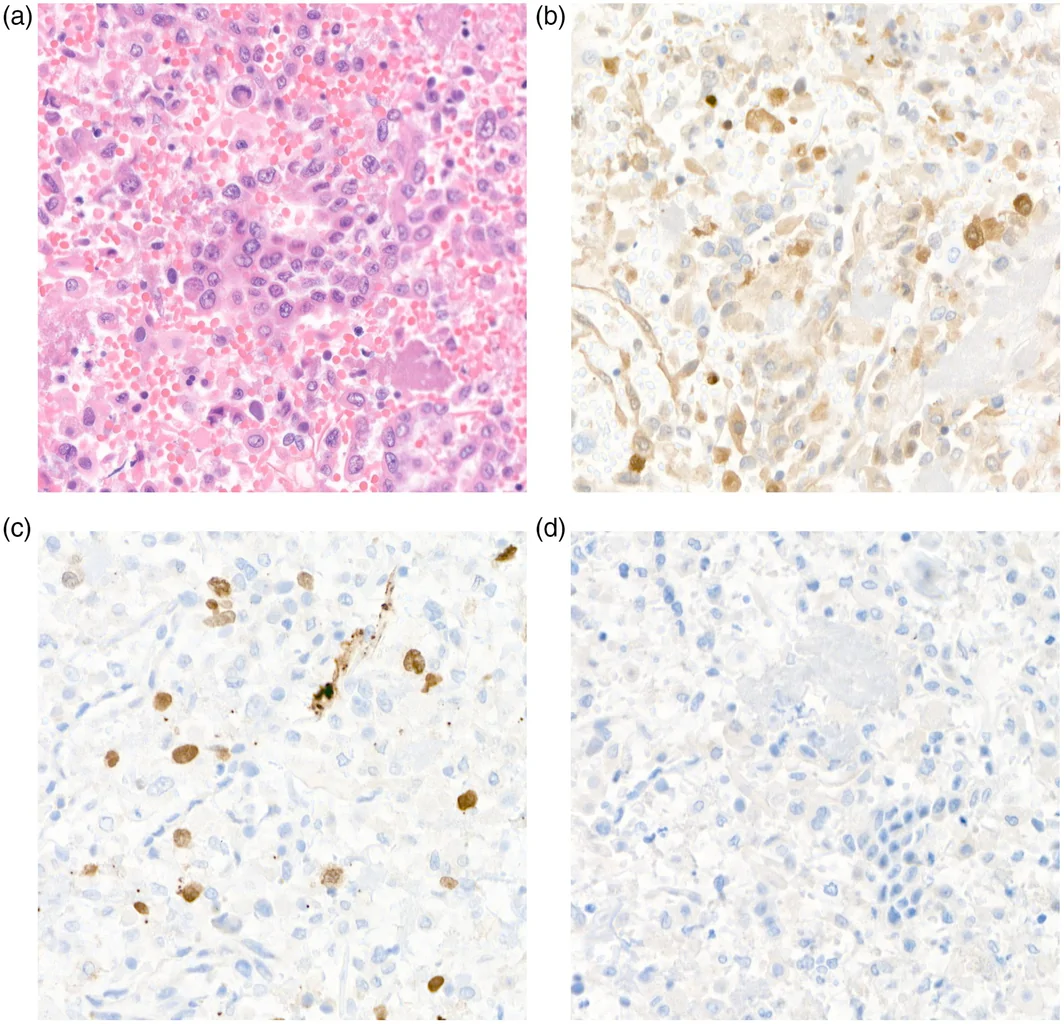
Cell block and immunocytochemistry. (a) Cell block, 40×, H&E. (b) S100, 40×. (c) SOX10, 40×. (d) Melan‐A, 40×. [Color figure can be viewed at wileyonlinelibrary.com]

**FIGURE 4:**
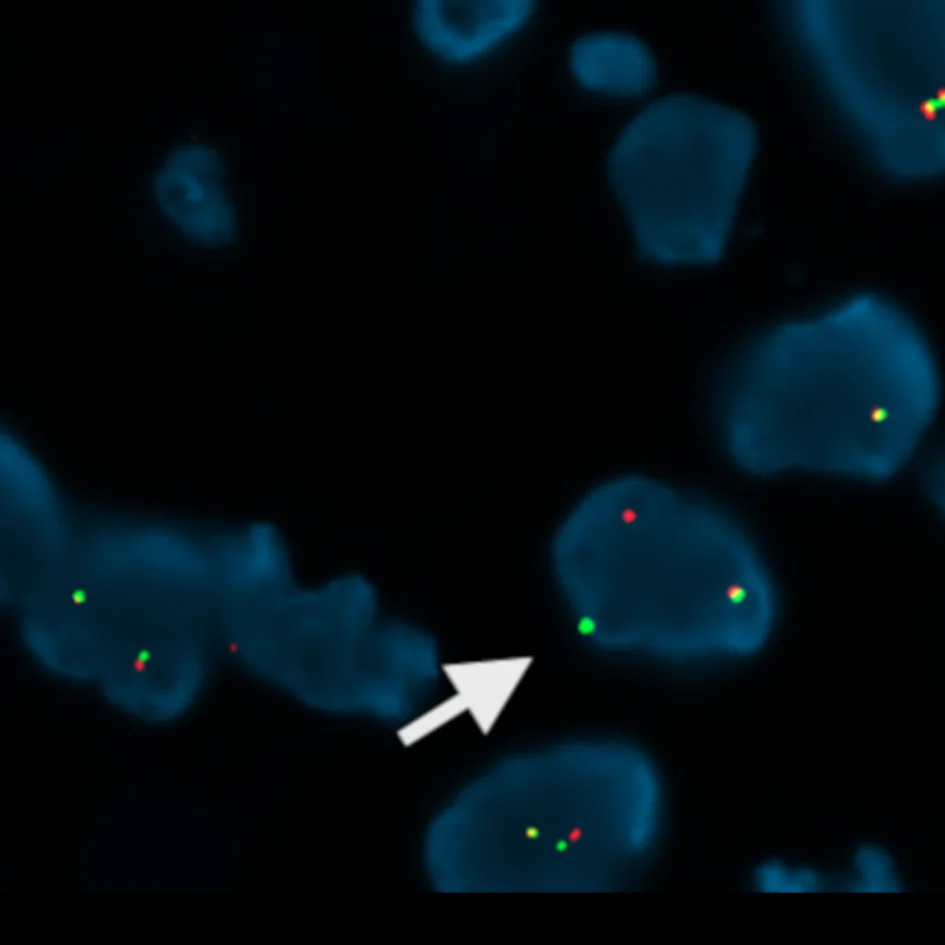
*ESWR1* break‐apart fluorescence in situ hybridization. The orange and green probes represented the centromere side, 5′ of the EWSR1 gene and the telomere side, 3′ of the EWSR1 gene respectively. Arrows: Two malignant cell nuclei showing unpaired signals. [Color figure can be viewed at wileyonlinelibrary.com]

The patient's sputum was also sent for cytological examination. Liquid‐based preparation of the sputum sample identified small numbers of suspicious cells in isolation and clusters with increased nucleus‐to‐cytoplasm ratio, hyperchromatic nuclei, irregular nuclear outline and prominent nucleoli (Figure 2). Pericardial and peritoneal effusion fluid were sent for liquid‐based and cell block preparation, which showed a moderate number of malignant cells in clusters and sheet‐like arrangement possessing a moderate amount of cytoplasm. The tumor nuclei were enlarged, hyperchromatic, irregular with prominent nucleoli, mostly a single nucleolus per nucleus and identified in most tumor nuclei. Nuclear irregularity was notable with folded, kidney bean and convoluted forms present (Figure 2). By immunohistochemistry on formalin‐fixed, paraffin embedded cell block preparation from pericardial effusion fluid, the malignant cells were patchily positive for S‐100 (Dako, Z0311) and SOX‐10 (Cell Marque, 383R‐16), and were negative for HMB‐45 (Dako, M634), Melan‐A (Dako, M719601) and PRAME (Abcam, ab219650) (Figure 3). Break‐apart fluorescence in situ hybridization (FISH) using ZytoLight SPEC EWSR1 Dual Color Break Apart Probe was performed on tissue section of formalin‐fixed paraffin embedded cell block prepared from pericardial effusion fluid. Neoplastic cellularity of 70% was noted in the marked area on the slide. A total of 50 interphase malignant cells nuclei were examined. There were isolated enlarged nuclei showing unpaired signals, suggesting the presence of gene rearrangement of the EWSR1 gene, at chromosome location 22q12.2, in some of the isolated malignant cells (Figure 4). These ancillary tests established the diagnosis of metastatic CCSST in the pericardial and peritoneal cavity. The patient received in‐patient palliative treatment and succumbed 2 months after admission.

## 3 Literature Review

Literature search using the Medical Subject Headings (MeSH) “clear cell sarcoma”, “cytology”, “pleural effusion”, “pericardial effusion”, “peritoneal effusion” and “sputum” retrieved six articles [9, 10, 11, 12, 13, 14] of interest describing 10 cases of CCSST with malignant effusion. With known data, the patients ranged from 19 to 54 years old and had equal gender distribution (5 males and 5 females). The primary site of most reported cases localised to the extremities (*n* = 8/10, 80%). None of the reported cases developed malignant effusion on first presentation. The time from first presentation to development of malignant effusion ranged from 2 months to 3 years. The most commonly involved body cavity by malignant effusion was the pleural cavity (*n* = 8/10, 80%), followed by pericardial (*n* = 2/10, 20%), pelvic (*n* = 1/10, 10%) and endobronchial/sputum (*n* = 1/10, 10%). The patients died of disease at 17 to 53 months after development of malignant effusion.

The cytomorphological features of CCSST in effusion fluid described in the reported cases, as well as what was encountered in the current case, were consistently malignant. The cytomorphology commonly reported in all series included malignant cells arranged in small clusters or in isolation, displaying a high nucleus‐to‐cytoplasm ratio, moderate‐to‐marked nuclear pleomorphism, and prominent nucleoli. Some series described other cytological features such as plasmacytoid morphology with eccentric nuclei or dense globoid cytoplasmic inclusions. However, these features were not consistently observed and were reported to be less frequent than in fine needle aspiration cytology [11, 12]. Notably, the presence of melanin pigment that is highlighted as a helpful diagnostic feature in histopathology was not encountered in all reported effusion cases and in our current case.

Including our current case and the reported cases in literature that included immunohistochemical studies, the CCSST cells in effusion fluid cell block demonstrated the following immunophenotype: S‐100 positive (*n* = 3/4), HMB‐45 positive (*n* = 2/3), SOX‐10 positive (*n* = 1), NSE positive (*n* = 1), Melan‐A negative (*n* = 1) and PRAME negative (*n* = 1). Molecular study was not performed in cases reported in the literature.

## 4 Discussion

Data extracted from the literature suggested a delayed onset malignant effusion in CCSST, in which the time from first presentation to development of malignant effusion ranges from 2 months to 3 years. Our current case may act as an outlier, in which malignant effusion developed after 29 years since first diagnosis. Once developed, CCSST with malignant effusion conferred a grave prognosis, in which the patients died of disease at 17 to 53 months after development of malignant effusion. Our current case demonstrated an even poorer prognosis, in which the patient died of disease at 2 months after his first admission for disease recurrence.

Sputum cytology was well established to be a low cost, non‐invasive screening tool for the early detection of respiratory pathology such as lung adenocarcinoma [15]. Sputum cytology was reported in one of the literature reviewed [12], as was performed in our current case. Both of the cases showed scanty yet sufficiently diagnostic suspicious or malignant cells in the sputum. This raises the possibility that in advanced disease, sputum cytology may have some diagnostic value for metastatic CCSST.

As an entity that required great diagnostic skills in the histopathology setting, CCSST posed an even greater challenge in effusion fluid cytological, as it is rare even in large retrospective series [16] The cytomorphology, while consistently suggesting a malignant behavior, was rather non‐specific in establishing the cellular lineage. The cytomorphology, while consistently suggesting a malignant behaviour, was rather non‐specific in establishing the cellular lineage. The cytological features in effusion significantly overlapped with metastatic carcinoma in terms of the cohesion, melanoma with prominent nucleoli and occasional binucleation and mesothelial neoplasms with sheet‐like architectural arrangements. Pathognomonic cytological features for diagnosis of CCSST were also lacking. Cytoplasmic melanin pigment that was deemed helpful when present in histopathology specimen was not identified in all reported cases of effusion involved by CCSST [16] (Table 2).

**TABLE 2:** Diagnostic features of clear cell sarcoma.

	Cytomorphology	Other cytologic features	Ancillary tests	
			Positive	Negative
Current report	Single prominent nucleoli Nuclear irregularity—folded, kidney bean, convoluted forms	Clusters Sheet‐like architecture	HMB‐45 S‐100 SOX‐10 *EWSR1* break‐apart FISH	Melan‐A PRAME
Parwani et al.	Single prominent nucleoli Binucleation Cytoplasmic globoid inclusion			
Su et al.	Binucleation and multinucleation	Clusters Tumor phagocytosis		
Creager et al.	Prominent nucleoli Intranuclear pseudoinclusions		HMB‐45, and NSE positive S‐100 Neuron specific enolase	
Caraway et al.	Prominent nucleoli. Cytoplasmic vacuolization	Karyorrhexis		
Nguyen et al.	Cytoplasmic and nuclear molding Cytoplasmic vacuolization Prominent and irregular nucleoli.	Clusters Sheets	S‐100 PAS‐positive cytoplasmic granules	
Keller et al.	Prominent nucleoli		HMB‐45	S‐100

To facilitate the diagnosis, immunohistochemistry might be performed on cell blocks made from effusion fluid if quantity allows. However, despite the small sample size, the immunophenotype of CCSST cells showed variability across different reported cases. Different melanocytic lineage markers were not consistently positive in the reported cases, and no single immunohistochemical marker can be diagnostic. In real‐life practice, it might be pragmatic to employ a panel of melanocytic markers to better characterise the CCSST cells.

The current case might shed new light on the confirmatory diagnosis of CCSST effusion by molecular technique. To our best knowledge, our reported case was the first in the literature that performed a molecular study on CCSST in effusion fluid, which demonstrated EWSR1 gene rearrangement through break‐apart FISH. In the ambiguity of cytomorphology and immunohistochemistry, triaging effusion fluid for cell block preparation followed by diagnostic molecular studies such as *EWSR1* break‐apart FISH and RT‐PCR for *EWSR1‐ATF1* fusion may offer more diagnostic confidence.

## 5 Conclusions

CCSST with malignant effusion proved to be a diagnostic challenge. The cytomorphology was malignant yet non‐specific, and correlation with clinical history and radiological findings was crucial to offer a high index of suspicion to triage the effusion fluid to cell block preparation, immunohistochemical studies and more diagnostic molecular tools, such as EWSR1 break‐apart FISH or RT‐PCR for EWSR1/ATF1 fusion.

## Author Contributions

W.H.W. and J.J.X.L. conceived the idea of the study. W.H.W., J.N.K.M., and J.J.X.L. collected and interpreted clinicopathological data. W.H.W., J.N.K.M., and J.J.X.L. reviewed the slides and prepared the figures. W.H.W. drafted the manuscript and J.J.X.L. critically revised the manuscript. All authors have read and approved the manuscript.

## Funding

The authors have nothing to report.

## Conflicts of Interest

The authors declare no conflicts of interest.

## Data Availability

Data sharing not applicable to this article as no datasets were generated or analysed during the current study.
